# The Austrian Remedy for Cholera

**Published:** 1851-10

**Authors:** William Herapath

**Affiliations:** London Lancet.


### ARTICLE XVIII.

### *The Austrian Remedy for Cholera.*

The chief commissioner of the Birmingham police lately placed in my hands a bottle of cholera medicine, which he had received from the head of the Austrian police, as being in use in that country under the influence of the government, it having been found to be a specific.

The printed description stated, "that the inventor had been allowed in 1831-2 by the government to try its efficacy on some convicted criminals, and that the result was the recovery in *every* instance; and that many thousand persons having since been cured by it, the government in 1849, commanded its employment in several large provinces, in prisons and public establishments, including the police, *and that not a single person who took the specific, where any hope of life remained, failed to*

*recover*, and that, too, without the slightest detriment to general health." This was apparently so decisive of its great value, that I determined to analyze it, and publish its components for the use of the world at large.

I find in the fluid ounce,—

| | Grains. |
|---|---|
| Sulphuric acid (density 1.845) . . . | 19. |
| Nitric acid (density 1.500) . . . . . | 12. |
| Sugar . . . . . . . . | 24. |
| Water . . . . . . . . . | 406.5 |
| Fluid ounce, specific gravity, . 1.055 = | 461.5 |

The sugar is now that of grapes, but it has no doubt been altered by the acids.

The mode of administration is said to be as follows :—

As soon as any premonitory symptoms of cholera show themselves, one tea-spoonful of the mixture is given, diluted with four or five of water ; cold water is also freely drunk immediately after. At the end of half an hour, in mild cases, the dose is repeated, and this is often all that is required.

The original anguish of the patient is generally found to undergo a remarkable diminution soon after the first dose ; he becomes warmer, and the pain in the stomach and breast rapidly abates, and then ceases. In the same degree in which the patient recovers his warmth, he becomes conscious of thirst, and feels a longing for cold water ; in order to satisfy this, a tea-spoonful of the acid is mixed with a pint of water, and of this the patient is allowed to take *ad libitum.*

When the symptoms do not rapidly yield, the dose is repeated at short intervals in the way already described. Where vomiting does not occur, four or five spoonfuls generally effect the cure. When, however, the phenomena of collapse have already set in, with continual vomiting, diarrhea, &c., the acid mixture must be given in double doses—namely, two table-spoonfuls at a time, in four or five of water, and repeated after every attack of vomiting until the sickness ceases, and the cramps abate ; after the vomiting has ceased, the medicine must still be repeated every quarter of an hour until at least six spoonfuls of the mixture have been retained on the stomach. Cases have occurred in which it has been necessary to give as many as ten to fourteen spoonfuls before vomiting ceased. Cessation of pain, abatement of cramp, and a warm perspiration, are the first signs of amendment. Should quiet sleep set in, it must not be interrupted. Cold water may be freely given, unless perspiration has commenced, when no more should be given than necessary to abate thirst. All warm or spirituous liquors should be avoided as so much poison.

This horrible complaint has hitherto baffled all practitioners, and eluded every mode of treatment that I have seen practiced; but this remedy comes with so good a character, and is so unlike those I have hitherto heard of, that I think it well worth a trial; nor can I refrain from mentioning that it has been remarked, that Asiatic cholera does not prevail in cider counties, where the general beverage has some resemblance to this medicine, though weaker in degree.

I am, sir, your obedient servant,
WILLIAM HERAPATH.
[*London Lancet.*

